# Biphasic Dynamics of Macrophage Immunometabolism during *Mycobacterium tuberculosis* Infection

**DOI:** 10.1128/mBio.02550-18

**Published:** 2019-03-26

**Authors:** Lanbo Shi, Qingkui Jiang, Yuri Bushkin, Selvakumar Subbian, Sanjay Tyagi

**Affiliations:** a Public Health Research Institute, New Jersey Medical School, Rutgers Biomedical and Health Sciences, Rutgers—The State University of New Jersey, Newark, New Jersey, USA; University of Texas Health Science Center at Houston

**Keywords:** immunometabolism, arachidonic acid metabolism, arginine metabolism, bioactive lipids, glycolysis, host-directed therapy, immune response, macrophage polarization, metabolic modulation, redox balancing

## Abstract

Macrophages are the primary targets of Mycobacterium tuberculosis infection; the early events of macrophage interaction with M. tuberculosis define subsequent progression and outcome of infection. M. tuberculosis can alter the innate immunity of macrophages, resulting in suboptimal Th1 immunity, which contributes to the survival, persistence, and eventual dissemination of the pathogen. Recent advances in immunometabolism illuminate the intimate link between the metabolic states of immune cells and their specific functions. In this review, we describe the little-studied biphasic metabolic dynamics of the macrophage response during progression of infection by M. tuberculosis and discuss their relevance to macrophage immunity and M. tuberculosis pathogenicity. The early phase of macrophage infection, which is marked by M1 polarization, is accompanied by a metabolic switch from mitochondrial oxidative phosphorylation to hypoxia-inducible factor 1 alpha (HIF-1α)-mediated aerobic glycolysis (also known as the Warburg effect in cancer cells), as well as by an upregulation of pathways involving oxidative and antioxidative defense responses, arginine metabolism, and synthesis of bioactive lipids. These early metabolic changes are followed by a late adaptation/resolution phase in which macrophages transition from glycolysis to mitochondrial oxidative metabolism, with a consequent dampening of macrophage proinflammatory and antimicrobial responses. Importantly, the identification of upregulated metabolic pathways and/or metabolic regulatory mechanisms with immunomodulatory functions during M1 polarization has revealed novel mechanisms of M. tuberculosis pathogenicity. These advances can lead to the development of novel host-directed therapies to facilitate bacterial clearance in tuberculosis by targeting the metabolic state of immune cells.

## Abstract (precis)

Macrophages are the primary targets of Mycobacterium tuberculosis infection; the early events of macrophage interaction with M. tuberculosis define subsequent progression and outcome of infection. M. tuberculosis can alter the innate immunity of macrophages, resulting in suboptimal Th1 immunity, which contributes to the survival, persistence, and eventual dissemination of the pathogen.

## INTRODUCTION

As essential components of innate immunity, macrophages play multiple roles in immune surveillance, defense against pathogens, and resolution of inflammation. The striking plasticity and diversity of macrophage functions are closely associated with their polarization state along the spectrum of M1 (classically activated macrophages) or M2 (alternatively activated macrophages) polarization (1, 2). M1 macrophages play essential roles in host defense through high-level expression of proinflammatory and antimicrobial molecules, such as interleukin-1β (IL-1β), IL-12, tumor necrosis factor (TNF), and inducible nitric oxide synthase/nitric oxide synthase 2 (iNOS/NOS2) (1, 3). M2 macrophages help maintain tissue homeostasis and control inflammation through high-level expression of anti-inflammatory cytokines, such as IL-4, IL-10, IL-13, and transforming growth factor β (TGF-β) (1, 4, 5). A growing body of evidence has revealed an intimate link between the polarization states of macrophages and their bioenergetic pathways (6, 7). M1 cells predominantly utilize aerobic glycolysis (similarly to the Warburg effect in cancer cells) for the generation of ATP and biosynthetic intermediates (6, 8). In contrast, M2 cells predominantly use mitochondrial oxidative phosphorylation and glutamine metabolism as major carbon and nitrogen sources, respectively (6–8), which is similar to what is seen with nonpolarized, resting macrophages. Comparative transcriptome analysis of macrophage polarization results from various host-pathogen interactions uncovered an upregulation of genes having a similar core M1 response signature (9), suggesting a general metabolic reprogramming of macrophages in response to infection.

Tuberculosis (TB), an infectious disease caused by Mycobacterium tuberculosis, is the leading cause of death from a single infectious agent, responsible for an estimated 1.3 million deaths in 2017 (10). After successful pulmonary infection, M. tuberculosis survives and proliferates within macrophages until expression of delayed Th1 immunity, which is associated with the formation of granulomas (11, 12), eventually limits the growth of the pathogen (13, 14). M. tuberculosis can also persist and exacerbate pathophysiological manifestations within granulomas, ultimately resulting in progression of infection and bacillary dissemination (14, 15). It is generally believed that suboptimal levels of proinflammatory and antimicrobial mediators during initial stages of the infection and an elevated inflammatory response during the chronic stage of the infection facilitate infection progression and completion of the pathogen infection cycle. A recent study of primary murine macrophage responses to M. tuberculosis infection performed with the high-throughput gene expression profiling platform called CAGE (cap analysis of gene expression) revealed a time-dependent transcription landscape (16). These data underscore the dynamic nature of host-pathogen interactions. In general, the early responses of primary macrophages or cell lines to M. tuberculosis infection are marked by core M1 polarization with shared expression patterns of genes that include those encoding receptors, signal transduction molecules, and transcription factors (9, 17–20). Parallel and comparative analyses of pathogen-specific responses in human primary macrophages identified M. tuberculosis-mediated inhibition of IL-12 production as an important mechanism for the survival of the pathogen in response to host defenses (19), which is consistent with the critical role of IL-12 in the development of protective immunity against M. tuberculosis (21, 22). Other mechanisms of M. tuberculosis defense against macrophage immunity include (i) prevention of phagolysosome maturation (23, 24); (ii) subversion of pathogen recognition by host immune cells and manipulation of macrophage recruitment (25); (iii) inhibition of host-protective cytokines (TNF, IL-12, IL-1β) with the induction of anti-inflammatory molecules such as IL-10 (26, 27); and (iv) the activation of bacterial resistance mechanisms, including induction of the DosR dormancy regulon (28, 29), shifting of bacterial respiratory pathways to anaerobic respiration (30), and a metabolic shift of bacterial carbon flux from the generation of biosynthetic precursors during growth to the formation of storage compounds, such as triacylglycerol during growth arrest (31, 32). However, our understanding of the metabolic characteristics of macrophages in response to M. tuberculosis infection and of whether any alteration of the metabolic state contributes to a suboptimal macrophage response is still very limited.

In this review, we describe the little-studied biphasic metabolic dynamics of macrophage responses to M. tuberculosis infection by systematically analyzing the metabolic patterns reported in representative transcriptome databases and/or the supplementary data files from studies of primary macrophage infection in the literature. Our analysis also identifies immunomodulatory metabolic pathways and mechanisms accompanying M1 polarization, revealing previously uncharacterized aspects of M. tuberculosis pathogenesis. We also discuss potential therapeutic intervention strategies to enhance protective antimicrobial responses of macrophages by targeting specific metabolic pathways.

## THE EARLY PHASE OF METABOLIC REPROGRAMMING

### The Warburg effect and its regulation.

We carried out differential gene expression analysis using the Web-based tool GEO2R (https://www.ncbi.nlm.nih.gov/geo/geo2r/), taking advantage of the transcriptome databases of C57BL/6 bone marrow-derived macrophages (BMDMs) up to 48 h after infection with M. tuberculosis H37Rv (20). We also profiled the differential host gene responses of B6D2F1 BMDMs following infection by clinical strains M. tuberculosis CDC1551 or HN878 (33). As noted previously (20, 33), functional characterization of the temporal gene expression signature of macrophages showed biphasic early upregulation of immune response genes (up to 8 h of infection), which is similar to the M1 transcriptional response signature seen upon M. tuberculosis infection in other studies (9, 17–19), followed by a late (from 24 to 48 h of infection) downregulation of immune response genes (17, 20, 33). This biphasic gene expression response landscape in C57BL/6 BMDMs, as elucidated with a recent high-throughput CAGE platform (16), underscores the dynamic nature of host-pathogen interactions during the progression of macrophage infection by M. tuberculosis.

**FIG 1:**
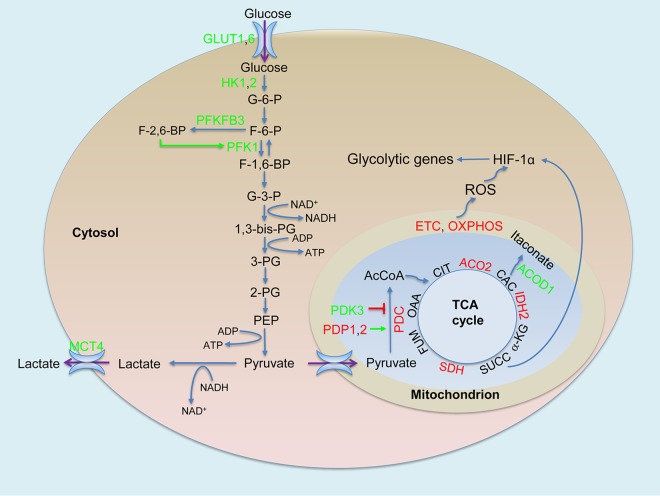
The Warburg effect in M. tuberculosis-infected macrophages. The early phase of macrophage infection is accompanied by increased glycolytic flux with consequent lactate formation and secretion and, concurrently, decreased mitochondrial oxidative metabolism exemplified by decreased pyruvate oxidation by the pyruvate dehydrogenase complex (PDC) and the downregulation of the TCA cycle and oxidative phosphorylation (OXPHOX). Increased levels of PFKFB3, one of the key regulatory mechanisms to promote glycolytic flux, together with elevated levels of glucose uptake transporters (GLUT1 and GLUT6) and other glycolytic enzymes, including hexokinases (HK1 and HK2), type 1 phosphofructokiase (PFK-1), and lactate secretion transporter member 4 (MCT4), illustrate the occurrence of the Warburg effect. The Warburg effect is associated with increased expression and activity of HIF-1α caused by the production of reactive oxygen species (ROS) derived from the respiratory electron transport chain (ETC) and OXPHOX and by the succinate (SUCC) accumulation from the TCA cycle in mitochondria. Green, increased expression/activity; red, decreased expression/activity; green arrow, stimulation, red block line, inhibition. Abbreviations: PDK3, pyruvate dehydrogenase kinase 3; PDP1-2, pyruvate dehydrogenase phosphatase 1 and 2; PDC, pyruvate dehydrogenase complex; ACO2, aconitase 2; ACOD1, aconitate decarboxylase 1; IDH2, isocitrate dehydrogenase 2; SDH, succinate dehydrogenase; G-6-P, glucose-6-phosphate; F-6-P, fructose-6-phosphate; G-3-P, glyceraldehyde-3-phosphate; 1,3-bis-PG, 1,3-bis-phosphoglycerate; 3-PG, 3-phosphoglyceride; 2-PG, 2-phosphoglyceride; PEP, phosphoenolpyruvate; AcCoA, acetyl CoA; OAA, oxaloacetate; CIT, citrate; CAC, cis-aconitate; α-KG, α-keto-glutarate; FUM, fumarate. Web-based GEO2R (https://www.ncbi.nlm.nih.gov/geo/geo2r/) was used for differential gene expression analysis, and changes described correspond to an adjusted *P* value of <0.05 at corresponding time points relative to uninfected controls. Microarray data are from the Gene Expression Omnibus (GEO) repository (www.ncbi.nlm.nih.gov/geo/; accession numbers GSE79733 and GSE31734).

Results of the present analysis, which focused exclusively on central metabolism, also show biphasic metabolic profiles that include an early phase (4 to 8 h postinfection) and a late adaptation/resolution phase (24 to 48 h postinfection). Our observations align with expression profiles of metabolic genes presented in the supplementary data file in the article by Roy et al. (16). The prevailing metabolic characteristic of the early phase of macrophage infection is glycolytic flux that is increased overall concurrent with downregulation of oxidative phosphorylation (Fig. 1), which is analogous to the Warburg effect in cancer cells (34). The state is manifested by upregulation of *Hif1a*, which encodes hypoxia-inducible factor 1 alpha (HIF-1α), which is responsible for the Warburg effect (35–38), and of genes encoding key enzymes of the Warburg effect, which include glucose uptake transporter 1 (GLUT1) and GLUT6; hexokinase 1 (HK1) and HK2; phosphofructokinase liver (PFKL) type from the three-member phosphofructokinase-1 (PFK-1) family; 6-phosphofructo-2-kinase/fructose-2,6-biphosphatase 3 (PFKFB3), an essential enzyme responsible for elevated glycolytic flux from the phosphofructokinase 2 (PFK-2) family; and the major lactate secretion transporter member 4 (MCT4 or SLC16A3). Using a murine model of low-dose, respiratory M. tuberculosis infection in C57BL/6 mice, we previously reported increased HIF-1α mRNA and protein levels and a metabolic remodeling of central metabolism similar to the Warburg effect in macrophages and T cells of infected mouse lungs (39), which coincides with expression of Th1 immunity (28). Recent studies confirmed a requirement for HIF-1α during the activation of Th1 immunity to control mycobacterial infection, including HIF-1α elicited by M. tuberculosis in mice (40, 41). In primary human and murine cells infected with M. tuberculosis, a shift toward aerobic glycolysis is also crucial for key innate immune functions, specifically, those mediated through IL-1β (42). Furthermore, we reported the occurrence of the Warburg effect and observed an upregulation of different glycolytic isozymes in a rabbit model of active TB and in lung granulomas of patients with active TB (43), which likely reflected the varied metabolic states of immune cells during the complex inflammatory responses in tuberculosis granulomas, such as coexistence of Th1 effector T cells and M1 macrophages with regulatory T cells and M2 macrophages. Multiple factors, including possible low-oxygen tension developed at late stages of granuloma development in TB, likely contribute to HIF-1α induction and the Warburg effect (discussed in reference 43), although the Warburg effect is independent of the status of oxygen tension. Accumulating evidence indicates that HIF-1α-mediated metabolic remodeling in immune cells is a general metabolic signature during inflammation and/or in response to various infections, including HIV infections (44–51), that is indispensable for meeting the bioenergetic need of effector functions of activated immune cells.

A key metabolic regulator of glycolytic flux is the metabolite fructose-2,6-bisphosphate (F-2,6-BP). This metabolite functions as a potent allosteric activator of glycolytic PFK-1 and an inhibitor of gluconeogenic fructose-1,6-bisphosphate (F-1,6-BP) phosphatases, thereby providing a mechanism for switching between the two opposing pathways (52, 53). F-2,6-BP is generated from fructose-6 phosphate (F-6-P) by PFK-2 family members, which include four bifunctional enzymes (PFKFB1 to PFKFB4) having both kinase and phosphatase activities (54). PFKFB3 has the highest kinase/phosphatase ratio (52) and plays an essential role in the metabolic switch to glycolysis in lipopolysaccharide (LPS)-treated murine peritoneal macrophages (8). The induction of only *Pfkfb3* among the PFK-2 family members during the early phase of murine macrophage M. tuberculosis infection is expected to favor the accumulation of F-2,6-BP, which would in turn promote glycolysis by inhibiting gluconeogenesis and stimulating the glycolytic activity of PFK-1 family members (Fig. 1), such as PFKL. Interestingly, much stronger induction of *Pfkfb3* in M. tuberculosis CDC1551-infected murine BMDMs in comparison to those infected by the hypervirulent M. tuberculosis HN878 (at 6 h postinfection) suggests a higher glycolysis rate in macrophages infected by M. tuberculosis CDC1551 (43), which may explain the higher levels of antimicrobial mediators observed in those macrophages (33).

The elevated glycolytic flux in M. tuberculosis-infected murine macrophages is in concert with an overall decreased oxidative metabolism in mitochondria (Fig. 1). This notion is supported by downregulation of genes encoding mitochondrial enzymes/proteins that include the pyruvate dehydrogenase complex (PDC) (E1 alpha [PDH-E1α], E1 beta [PDH-E1β], E2 [DLAT], component X [PDHX]), tricarboxylic acid (TCA) cycle enzymes (aconitase 2 [ACO2], isocitrate dehydrogenase 2 [IDH2], succinate dehydrogenase complex subunits [SDHA, SDHC, SDHD]), and multiple components of respiratory chain complexes that include NADH dehydrogenase, cytochrome *c* reductase, and H^+^ transporting ATPase. Oxidative decarboxylation of pyruvate by mitochondrial PDC links glycolysis to the TCA cycle, a process that is regulated via phosphorylation (inactivation) by a family of pyruvate dehydrogenase kinase 1 (PDK1) to PDK4 and dephosphorylation (activation or reactivation) by pyruvate dehydrogenase phosphatase 1 (PDP1) and PDP2 (55). During the early phase of murine macrophage infection by M. tuberculosis, the two mechanisms appear to function in concert to inhibit pyruvate oxidation in mitochondria, thereby promoting carbon flux through glycolysis. This coordination is achieved by (i) an upregulation of only *Pdk3*, whose product has the highest activity among the four PDKs for inhibiting PDC activity and is inhibited little by high concentrations of pyruvate (56, 57), and (ii) a downregulation of *Pdp1* and/or *Pdp2*, which prevents PDC from activation via dephosphorylation (Fig. 1).

The elevated glycolytic flux is also attributed, at least in part, to the accumulation of the TCA cycle intermediate succinate, which functions as a metabolic signal to link metabolism and immunity (58, 59). Succinate boosts HIF-1α activity by inhibiting HIF-α prolyl hydrolases, drives IL-1β production, and limits the anti-inflammatory responses in activated macrophages (59, 60). The succinate-promoted proinflammatory response appears to rely on the activity of SDH, as inhibition of SDH activity by dimethyl malonate through rapid hydrolysis to malonate, which is a potent inhibitor of succinate oxidation by SDH, abrogates the activity of LPS-induced IL-1β and boosts IL-10 production in BMDMs from C57BL/6 mice (59, 61). In M. tuberculosis-infected murine macrophages, we observed downregulation of *Sdh*, which likely contributes to the accumulation of succinate, thereby leading to the induction of HIF-1α, the Warburg effect, and the proinflammatory responses.

Recent studies using BMDMs from C57BL/6 wild-type (WT) and *Irg^−/−^* mice indicated that SDH activity is also regulated by itaconate (62, 63), a metabolite that is derived from the TCA cycle intermediate cis-aconitate, by increased activity of aconitate decarboxylase 1 (ACOD1) (64). The metabolic breakpoint of the TCA cycle, caused by the downregulation of mitochondrial *Idh2*, is responsible for redirecting the carbon flow from precursor isocitrate toward the formation of itaconate (7, 64). The *Acod1*, also known as immune-responsive gene 1 (*Irg1*), is highly upregulated in M. tuberculosis-infected murine macrophages and mouse lungs (per the transcriptome data in the work by Kang et al. [65] and our observations), as seen in ANA-1 macrophages treated with proinflammatory cytokines and Toll-like receptor (TLR) agonists, as well as in the spleen and lungs of mice infected with Listeria monocytogenes or Toxoplasma gondii (66). Itaconate has antimicrobial properties through inhibition of isocitrate lyase, the key enzyme of the glyoxylate shunt, a pathway essential for the growth of bacteria, including M. tuberculosis, under specific conditions (64). Itaconate was recently shown to inhibit SDH activity and increase levels of succinate. Surprisingly, it also modulates the macrophage proinflammatory response, as *Irg1^−/−^* BMDMs from C57BL/6 mice sustain higher HIF-1α mRNA and protein levels and produce more proinflammatory cytokines and antimicrobial molecules, such as IL-12, IL-1β, and NO, under proinflammatory conditions (62). These apparently opposing mechanisms, corresponding to the *Irg1*-mediated anti-inflammatory response and the succinate-mediated proinflammatory process during macrophage activation, appear to prevent damage to host cells that would otherwise occur as a consequence of a potentially prolonged state of hyperinflammation. It is not clear whether the anti-inflammatory effect from the robust *Irg1* upregulation is related to the simultaneous induction of anti-inflammatory cytokine IL-10, which is associated with M1 polarization at the early phase of M. tuberculosis infection (67).

### Increased oxidative stress and antioxidative defense responses.

**FIG 2:**
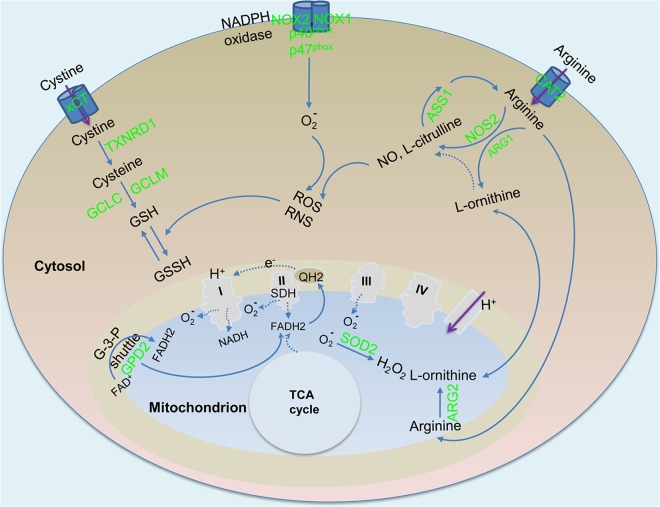
Redox balancing in M. tuberculosis-infected macrophages. ROS and reactive nitrogen species (RNS) were derived from (i) increased levels of NOS2 and NADPH oxidase; (ii) complexes of ETC due to an overall decrease in the levels of ETC and OXPHOX; and (iii) a process of reverse electron transport to complex I from reduced ubiquinone pool (QH2). FAD-dependent reactions contribute to reduction of the ubiquinone pool by increased level of mitochondrial glycerol-3-phosphate dehydrogenase 2 (GPD2) in the glycerol-3-phosphate (G-3-P) shuttle and by succinate dehydrogenase (SDH) of the respiratory complex II. The antioxidative defense response is mediated by increased mitochondrial superoxide dismutase 2 (SOD2) and glutathione (GSH) synthesis by glutathione synthase (GCLC and GCLM). The substrate cysteine used for the synthesis of GSH originates from reduction of the oxidized form of cystine (such as by thioredoxin reductase 1 [TXNRD1]), which is taken up extracellularly by increased xCT transporter. Increased levels of arginases, especially ARG2, diminish NO production by competing with NOS2 for the common substrate arginine, which is derived predominantly from increased levels of the arginine transporter (CAT2). Arginine can be regenerated by the activity of argininosuccinate synthetase 1 (ASS1) from products of arginine catabolism, such as l-citrulline. Green, increased expression/activity. Abbreviation: GSSH, oxidized glutathione. Data analysis was performed as described for Fig. 1.

Given the rapid increase in production of reactive oxygen species (ROS) and reactive nitrogen species (RNS) during M1 polarization and the potential deleterious effects of these molecules on cellular macromolecules, an increased level of oxidative stress and the subsequent antioxidative defense responses are important components of the metabolic remodeling (Fig. 2). Sources of oxidative stress derive from multiple metabolic processes. One is the production of ROS and RNS from the respiratory burst and upregulation of cytosolic iNOS/NOS2. NADPH oxidase complex mediates the respiratory burst by transferring electrons from NADPH to molecular oxygen with the formation of O_2_^-^ and/or of H_2_O_2_. In response to M. tuberculosis infection, *N*os2 is highly induced in murine macrophages, consistent with its essential role in mediating the antimicrobial response to M. tuberculosis (68). In contrast, induction of genes encoding the active NADPH oxidase complex, including cytosolic components (p40^phox^ and p47^phox^) and a cell membrane component (gp91^phox^ [NOX2] or NOX1), is moderate. NO resulting from iNOS/NOS2 activity can react with O_2_^-^ to form more-potent RNS such as ONOO^-^. Another source of ROS production is decreased oxidative phosphorylation in mitochondria. Specifically, inhibition of the electron transport chain (ETC) and mitochondrial function, which is likely caused by NO production from iNOS/NOS2 (69, 70), can lead to decreased redox (NAD^+^/NADH) and overproduction of ROS from the ETC, as seen in activated BMDMs from C57BL/6 mice (61). A third source of ROS production is the upregulation of mitochondrial glycerol-3-phosphate (G-3-P) dehydrogenase 2 (GPD2). As an important enzyme in the G-3-P shuttle, GPD2 oxidizes G-3-P, an intermediate of glycolysis that is imported into mitochondria from the cytosol, to dihydroxyacetone phosphate (DHAP) coupled with FAD-dependent reactions to funnel electrons into the coenzyme Q pool within the inner membrane of mitochondria (71). The GPD2-mediated reaction with an elevated reduction state of the coenzyme Q pool during electron transfer has been reported to serve as the source of mitochondrial ROS, which acts as an important signal during T cell activation (72, 73). The strong induction of *Gpd2* in M. tuberculosis-infected murine macrophages suggests the presence of an active G-3-P shuttle, probably contributing to ROS production during macrophage activation. Finally, the activity of SDH serves as another source of ROS. In addition to being a TCA enzyme, SDH serves as complex II of the respiratory ETC, and oxidation of succinate couples with a FAD-dependent reaction to funnel electrons to the coenzyme Q pool (74). A recent study indicated that succinate oxidation by SDH in the ETC and increased membrane potential due to elevated glycolysis lead to the production of mitochondrial ROS, which drives proinflammatory responses in BMDMs from C57BL/6 mice (61). Furthermore, the formation of ROS during succinate oxidation is probably due to reverse electron transfer to complex I (61, 75) (Fig. 2). In summary, these observations are consistent with the notion that mitochondria are the main source of ROS production during M1 polarization (75).

O_2_^-^ is detoxified via the concerted activities of superoxide dismutases and catalase. During M. tuberculosis infection of murine macrophages, *Sod1*, which encodes the cytosolic dismutase, shows a moderate decrease, while *Sod2*, which encodes the mitochondrial enzyme, is strongly induced. These observations are in agreement with the conclusion that mitochondria represent the main source of ROS (75). H_2_O_2_ generated from dismutation is further detoxified by increased catalase levels in M. tuberculosis-infected murine macrophages. Given the increased requirement for NAD^+^ to maintain an elevated glycolysis rate and the decreased capability of mitochondrial phosphorylation to oxidize NADH, increased NAD^+^ synthesis in activated immune cells serves as a mechanism for maintaining redox homeostasis. Nicotinamide phosphoribosyltransferase (NAMPT), a key enzyme involved in NAD^+^ synthesis, is increased in activated immune cells (76–78). *Nampt* is strongly induced in M. tuberculosis-infected murine macrophages, indicating the presence of an active redox-balancing process.

Another mechanism of antioxidative defense is mediated by production of intracellular glutathione (GSH), a major low-molecular-weight antioxidant that plays a crucial role in maintaining cellular redox balance by mediating the antioxidative response. Similarly to gamma interferon (IFN-γ) and LPS-activated BMDMs from CBA mice (79), there is increased GSH synthesis in M. tuberculosis-infected murine macrophages, as indicated by increased expression of *Gclc* and *Gclm*, which encode, respectively, the catalytic and modifier subunit of glutamate-cysteine ligase, an enzyme that catalyzes the first rate-limiting step of GSH synthesis (80). Given the typically low concentration of cysteine in the cytosol, the potential rate-limiting factor in GSH synthesis is related to the availability of cysteine as a substrate (81). Thus, import of cystine (an oxidized form of cysteine) from the extracellular environment, which is mediated by antiporter system xC^−^, consisting of xCT (also known as SLC7A11) and its chaperone CD98 (SLC3A2) (82), has been regarded as an important process for maintaining intracellular GSH levels and redox balance. The antiporter system xC^−^ mediates the uptake of extracellular cystine coupled to glutamate efflux (83). Activated macrophages from *xCT*-deficient C57BL/6 mice during tumorigenesis display impaired survival, and an xCT deficiency augments inflammation at the inflammation site, indicating a critical role for xCT in antioxidative defense response (84). The strong induction of *xCT* in M. tuberculosis-infected murine macrophages and mouse lungs (per the transcriptome data in the work by Kang et al. [65] and our observations), suggests a role of xCT in protecting host cells from oxidative stress and probably in prolonging the survival of infected macrophages, which would ultimately and inadvertently benefit the survival of the pathogen. Indeed, findings from a recent study indicate that increased *xCT* expression is associated with development of active TB in humans, and that *xCT*-deficient C57BL/6 mice have an enhanced ability to control M. tuberculosis growth with decreased bacillary load and reduced pathology in lungs in comparison to wild-type mice (85). Based on these observations, it appears reasonable to determine whether specific xCT inhibitors, such as sulfasalazine (SASP) (86), an anti-inflammatory drug routinely used in clinical therapy, can be used as host-directed therapy (HDT) to boost host antimicrobial response during TB treatment.

### Increased arginine uptake and metabolism: competition between two opposing activation states.

Given that arginine, the substrate of iNOS/NOS2 for the production of NO and other RNS, also serves as a substrate for arginase, balancing arginine metabolism between the two competing pathways constitutes an important regulatory mechanism in the polarization states of macrophages (87, 88) (Fig. 2). Indeed, increased arginine uptake and metabolism accompany macrophage activation with respect to both the M1 and M2 phenotypes (89). In M1 macrophages, arginine is required for synthesis of proteins, production of NO, and microbicidal activities, whereas in M2 macrophages, arginine is required for generation of polyamines and proline. The availability of intracellular arginine is a rate-limiting step for arginine metabolism, and arginine uptake by cationic amino acid transporter family member 2 (CAT2), encoded by *Slc7a2*, plays a critical regulatory role during murine macrophage polarization (89).

Two arginase isoforms (cytosolic type 1 [ARG1] and mitochondrial type 2 [ARG2]), which are encoded by different genes in mammals, have different tissue, cellular, and subcellular distributions (90, 91). ARG1 is mainly induced by Th2 cytokines in murine myeloid cells, dendritic cells, and granulocytes (88, 92, 93). ARG1-mediated inhibition of NO production from iNOS/NOS2 has been regarded as a mechanism of M. tuberculosis immune evasion. *Arg1*^flox/flox^;*Tie2*cre mice with an *Arg1* deficiency in macrophages had decreased M. tuberculosis numbers in lungs compared to control mice, and *Arg1*-deficient macrophages had an increased ability to kill M. tuberculosis relative to wild-type macrophages (94). In human TB granulomas, furthermore, there is a distinct spatial distribution consisting of M1 macrophages expressing iNOS at high levels and ARG1 at low levels in the central granuloma regions containing M. tuberculosis-infected macrophages compared to peripheral granuloma regions containing M2 macrophages, which have high expression of ARG1 and low expression of iNOS (95). However, the role of ARG1 in mediating immune cell functions appears to depend on the specific granuloma microenvironment. As reported in a murine TB granuloma model of dermal infection in *Nos2*-deficient C57BL/6 mice, which recapitulates several features of human TB granuloma pathology, such as hypoxia and caseation at the center of granulomas, ARG1 expression in macrophages of hypoxic granulomas plays a beneficial role in controlling bacterial growth and preventing lung pathology (96). Thus, the function of ARG1 appears to depend on the stage of infection. At initial stages of infection, ARG1 activity favors survival of the pathogen by dampening macrophage immunity through substrate competition with iNOS/NOS2; during the chronic stage of infection, when prolonged hyperinflammation is associated with exacerbation of lung immunopathology, ARG1 contributes to control of infection by modulating the development of lung immunopathology through repressing T cell proliferation, as seen in M. tuberculosis-infected *Nos2*-deficient mice (96). Impairment of T cell proliferation is attributable in part to ARG1-mediated depletion of l-arginine in the local extracellular microenvironment and the production of polyamines with anti-inflammatory functions (96).

In contrast to ARG1, information about the expression and function of mitochondrial ARG2 in immune cells remains scant. ARG2 accounts for more than 90% of arginase activity in LPS-activated RAW 264.7 cells (97). Unlike *Arg1*, *Arg2* is not significantly affected by Th1 or Th2 cytokine treatment of primary murine macrophages from various mouse strains, including the AKR/N, C57BL/6, and B6D2F1 strains (92, 98, 99). ARG2 is also the dominant isoform in dendritic cells, and its repression by the highly induced microRNA-155 during dendritic cell maturation is crucial for dendritic cells to drive T cell activation by controlling the availability of arginine in the extracellular environment (100).

Moderate induction of *Arg1* during the early phase of murine macrophage infection by M. tuberculosis, relative to the strong concurrent upregulation of *Cat2* (*Slc7a2*) and *Nos2*, suggests a limited role of ARG1 in dampening NO production from NOS2 during M1 polarization. In contrast, there is strong upregulation of mitochondrial *Arg2* in M. tuberculosis-infected murine macrophages. *Arg2*, along with *Nos2* and *Cat2*, is also induced during the expression of Th1 immunity in M. tuberculosis-infected mouse lungs (101) (per the transcriptome data in the work by Kang et al. [65] and our observation). A recent study characterizing the dynamic immune response landscape of M. tuberculosis-infected BMDMs from C57BL/6 mice also revealed strong *Arg2* upregulation during M1 polarization (see the supplemental data in reference 16). The observation that *Arg2* upregulation is associated with M. tuberculosis infection, but not with Th1 or Th2 cytokine pretreatment (see the supplemental data in reference 16), points to a more significant role of ARG2 relative to ARG1 in modulating macrophage immunity. Furthermore, in a murine model of gastritis caused by Helicobacter pylori, deletion of *Arg2* in C57BL/6 mice was found to be associated with enhanced M1 polarization, including increased NO production, and with improved control of H. pylori infection, indicating that ARG2 contributes to immune evasion of H. pylori by restricting M1 macrophage activation (102, 103).

The effects of *Arg2* expression on innate and adaptive immune responses are associated at least in part with liver X receptors (LXRs) LXRα and LXRβ, which serve as transcription regulators of genes involved in innate immunity and cholesterol metabolism (104–106). Activation of LXRs induces immunomodulatory functions in macrophages by inhibiting the expression of a cluster of genes involved in inflammation and innate immune responses, such as *Nos2* and *Il6*; some of these actions are mediated through LXR antagonism of NF-κB activity (107–109). Given that *Arg2* is also directly regulated by LXRs in primary macrophages from C57BL/6 mice and in RAW 264.7 cells (110), increased *Arg2* expression appears to contribute to anti-inflammatory functions of LXRs by controlling excess NO production during inflammation (110). Moreover, forced expression of ARG2 mimics the inhibitory effect of LXR activation on macrophage NO production, whereas inhibition of arginase activity partially reverses the inhibitory effect of LXR agonists on NO production in murine peritoneal macrophages or in RAW 264.7 cells (110, 111). Consistent with upregulation of *Arg2*, the inducible isoform LXRα-encoding *Nr1h3* is induced during the early phase of murine macrophage infection by M. tuberculosis. However, it was also reported in *Lxra*^–/–^, *Lxrb*^–/–^, and *Lxra*^–/–^*Lxrb*^–/–^ C57BL/6 mouse models of airway infection that LXR signaling, especially that mediated by LXRα, is associated with a protective immune response against M. tuberculosis, which is probably mediated by the IL-23/IL-17 axis with a contribution of neutrophils and Th17 cells (112). Further in-depth studies are needed to dissect the role and mechanism of ARG2 upregulation in mediating the metabolic state and function of host immune cells in TB.

### Increased turnover of long-chain fatty acids from phospholipids and synthesis of bioactive lipid mediators.

Production of inflammatory mediators during macrophage polarization requires increased synthesis of fatty acids and phospholipids. While there is a general slowdown of TCA cycle activity and oxidative phosphorylation, accumulation of the TCA cycle intermediate citrate, probably as a result of decreased levels of mitochondrial IDH2 and the metabolic breakpoint between isocitrate and α-ketoglutarate (7), plays a role in mediating inflammation (75, 113). Citrate is transported from mitochondria to the cytosol by a citrate carrier (*SLC25A1*), and its cleavage products are required for fatty acid synthesis and production of ROS, NO, and prostaglandins (113, 114). Knockdown of *SLC25A1* or inhibition of SLC25A1 activity leads to a significant decrease in the production of proinflammatory mediators in human primary macrophages and in U937 cells (113). Moreover, members of the acyl-coenzyme A (acyl-CoA) synthase long-chain family, especially ACSL1 and ACSL4, which are responsible for esterifying long-chain free fatty acids to intracellular fatty acyl-CoA, play important roles in phospholipid turnover and innate immunity (115–117). *Acsl1* is induced in the bone marrow and peritoneal macrophages of C57BL/6 mice after exposure to Gram-negative bacteria or to LPS or other proinflammatory molecules (116). *ACSL1/Acsl1* is also induced in monocytes from humans and mice with type 1 diabetes, and its deficiency in myeloid-specific *Acsl1*-deficient mice leads to decreased expression of proinflammatory cytokines, including IL-1β and TNF (115). In contrast to its function in β-oxidation in insulin target tissues, *Acsl1* is required for phospholipid turnover and promotes the synthesis of 16:0-CoA, 18:1-CoA, and 20:4-CoA in activated BMDMs or peritoneal macrophages from C57BL/6 mice; its deficiency reduces the turnover of several phospholipids containing these fatty acids (116). In particular, the role of *Acsl1* in phospholipid metabolism is likely associated with the generation of arachidonic acid (AA) metabolism-derived bioactive lipid mediators (117–119). Indeed, ACSL1, together with ACSL4, controls the turnover of AA-containing phospholipids (117). As a 20-carbon, unsaturated fatty acid distributed throughout the lipid bilayers of the cell, AA is usually esterified at the sn-2 position of glycerophospholipids. Cytosolic phospholipase A2 (cPLA2) catalyzes the release of AA from the sn-2 position of membrane glycerophospholipids (120, 121), thus making it available for functioning in the regulation of phospholipid acyl turnover for membrane maintenance or during the production of inflammatory lipid mediators (121–123). Following its production by cPLA2, AA is metabolized for the generation of bioactive eicosanoids such as prostanoids, leukotrienes, and lipoxins.

Prostanoids, especially the prostaglandin E series, are produced by consecutive actions of cyclooxygenases (COX1 and COX2) and prostaglandin synthases (124, 125). Leukotrienes and lipoxins are metabolites generated by lipoxygenases (LOXs) (126–128). Accumulating evidence indicates that the balance between prostaglandin E2 and lipoxin 4 is critical for controlling tuberculosis immunopathology (129, 130). Prostaglandin E2 plays a critical role in the inhibition of M. tuberculosis replication, as evidenced by prostaglandin E synthase-deficient (*Ptges*^−/−^) C57BL/6 mice harboring significantly higher bacterial burdens than wild-type mice (129). In contrast, lipoxin (e.g., lipoxin A4) production by the 5-lipoxygenase (ALOX5)-dependent pathway correlates with susceptibility of the host to M. tuberculosis infection (131). M. tuberculosis-infected *Alox5^−/−^* (C57BL/6, 129S Alox-5, F2/J) mice have no detectable lipoxin A4 in the sera, and they show an increased Th1 response with lower bacterial burden than wild-type (C57BL/6, 129 J F2) mice after pulmonary infection. Moreover, administration of a stable lipoxin 4 analog to M. tuberculosis-infected *Alox5*^−/−^ mice reverses the protection of *Alox5*^−/−^ mice (131).

**FIG 3:**
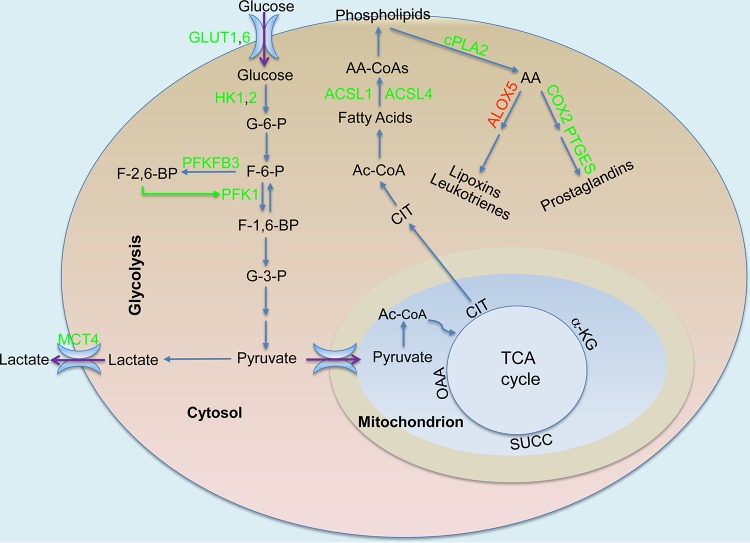
Production of bioactive lipids in M. tuberculosis-infected macrophages. The TCA cycle intermediate citrate (CIT) serves as precursor for the synthesis of fatty acids, phospholipids, and bioactive lipid mediators such as prostaglandins. Increased levels of acyl CoA synthase long-chain family members, especially ACSL1 and ACSL4, lead to increased incorporation of long-chain fatty acids, especially arachidonic acid (AA), into membrane phospholipids. Increased levels of cytosolic phospholipase A2 (cPLA2), which has a preference to release AA, result in high intracellular concentrations of AA, which serves as a substrate for the production of prostaglandins mediated by elevated levels of cyclooxygenase 2 (COX2) and prostaglandin E synthase (PTGES). Green, increased expression/activity; red, decreased expression/activity; green arrow, stimulation. Data analysis was performed as described for Fig. 1, and other abbreviations are as defined in the Fig. 1 legend.

The early phase of murine macrophage infection by M. tuberculosis is accompanied by overall increased levels of synthesis and turnover of long-chain fatty acyl-CoAs and increased production of prostaglandins (Fig. 3). This is evidenced by increased expression of *Acsl1*, *Acsl4*, *Pla2g4a* (*cPla2*), *Ptges*, and *Ptgs2* (also known as *Cox2*, encoding prostaglandin-endoperoxide synthase 2 [COX2]). This observation is also supported by findings revealing decreased production of lipoxins and/or leukotrienes, as indicated by concurrent downregulation of *Alox5* in M. tuberculosis-infected murine macrophages. Interestingly, hypervirulent M. tuberculosis strain HN878 appears to manipulate host cell lipid metabolism and modulate the production of bioactive lipid mediators such as prostaglandin E2. For example, *Fasn*, which encodes fatty acid synthase (FASN), a key enzyme involved in fatty acid synthesis, is induced in B6D2F1 BMDMs infected by M. tuberculosis HN878 but not in BMDMs infected by M. tuberculosis strain CDC1551 or strain H37Rv (20, 33). Moreover, relative to the M. tuberculosis CDC1551 strain, infection of BMDMs by M. tuberculosis HN878 leads to more induction of *Acsl4*, whose product has the most preference for arachidonate and eicosapentaenoate among C_8_-C_22_ saturated fatty acids and C_14_-C_22_ unsaturated fatty acids (132). This is in contrast to the expression of *cPla2*, which shows induction in CDC1551-infected BMDMs that is more robust than that seen in BMDMs infected by M. tuberculosis HN878 and whose product cytosolic phospholipase A2 has a marked preference for AA liberation from cellular phospholipids (123). Thus, consistent with previous observations (129), our analysis points to the conclusion that the hypervirulent M. tuberculosis HN878 strain induces diminished production of prostaglandin E2 in host cells due to the relatively high expression of *Acsl4* but low expression of *cPla2*. The result is a marked increase of AA incorporation into phospholipids and thus a reduced cellular level of free AA available for production of lipid mediators, including prostaglandin E2 (133). Prostaglandin E2 protects host cells from necrosis by preventing mitochondrial inner membrane instability and plasma membrane disruption (118, 119); thus, M. tuberculosis is able to evade macrophage defense in part by inhibiting prostaglandin E2 production and subsequent membrane repair (129). Control of M. tuberculosis infection by Th1 immunity in mouse lungs is also accompanied by increased expression of genes involved in phospholipid acyl-CoA turnover and prostaglandin E2 synthesis, including *Acsl1*, *cPla2*, *Cox2*, and *Ptges* (per the transcriptome data in the work by Kang et al. [65] and our observations). Taken together, the data revealing enhanced synthesis of long-chain fatty acyl-CoAs and their turnover into phospholipids with increased prostaglandin synthesis during the early phase of macrophage M. tuberculosis infection are consistent with M1 polarization (Fig. 3).

## THE ADAPTATION/RESOLUTION PHASE OF METABOLIC REPROGRAMMING

**FIG 4:**
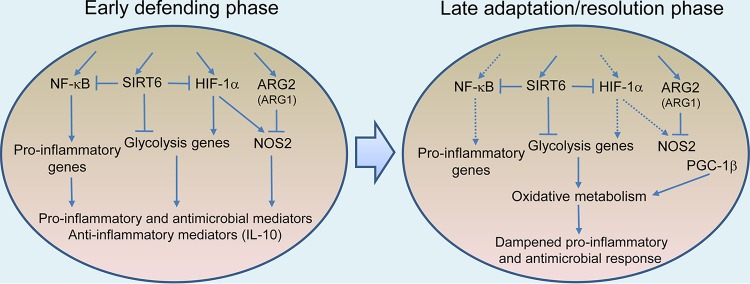
Metabolic dynamics and macrophage polarization during M. tuberculosis infection. The early phase of macrophage infection is accompanied by a metabolic switch from mitochondrial oxidative metabolism to HIF-1α-mediated Warburg effect and NOS2 induction and to activation of NF-κB-mediated proinflammatory responses. The concurrently increased levels of SIRT6 and ARG2, which may inhibit, respectively, the Warburg effect and NOS2 function, result in a suboptimal M1 response. Increased levels of PGC-1β, a key transcription factor in promotion of mitochondrial biogenesis and oxidative metabolism, coupled with a decreased Warburg effect, mark the transition to the late adaptation/resolution phase of macrophage infection with the consequent dampened proinflammatory response, which promotes M. tuberculosis survival and/or growth. Solid arrow, induction/activation; dotted arrow, dampened induction/activation; block line, inhibition. Data analysis was performed as described for Fig. 1.

The aforementioned metabolic reprogramming during the early phase of infection serves as a prerequisite for the production of antimicrobial and proinflammatory effector molecules of M1 macrophages. Moreover, the unchanged or decreased expression of genes involved in glutamine metabolism in these cells, which is associated with M2 polarization (7), is also in line with M1 polarization. This metabolic reprogramming accompanying M1 polarization is consistent with decreased survival of M. tuberculosis in C57BL/6 BMDMs during the early phase of infection (32). However, the simultaneous induction of several other metabolic pathways, such as the strong induction of *Arg2* and *Irg1*, which is expected to diminish antimicrobial and proinflammatory responses, also represents mechanisms of M. tuberculosis interference with the expression of macrophage immunity. This modulation of host cell metabolism to favor the survival and persistence of M. tuberculosis is evidenced by a simultaneous strong induction of anti-inflammatory cytokines, such as IL-10 (67), concomitantly with the M1 response (Fig. 4). As infection progresses to 24 and 48 h postinfection, the metabolic state of infected macrophages is characterized by an expression profile marked by an overall dampening of glucose uptake and glycolysis and a recovery of the TCA cycle activity and oxidative phosphorylation function. In particular, this shift in metabolic state is manifested by the induction of *Pgc1b*, which encodes peroxisome proliferator-activated receptor gamma (PPAR-γ)-coactivator-1β (PGC-1β), a key transcription factor in mitochondrial biogenesis that promotes oxidative metabolism (134, 135). In C57BL/6 BMDMs, PGC-1β transcriptionally regulates the development of M2 polarization by enhancing oxidative metabolism and mitochondrial biogenesis, thereby serving as a link that integrates macrophage metabolism and immune function (135). Transgenic expression of PGC-1β primes M2 activation and strongly inhibits proinflammatory cytokine production, whereas inhibition of oxidative metabolism or RNA interference (RNAi)-mediated knockdown of PGC-1β attenuates this immune response in HOXA9-ER cell lines (135). This shift in metabolic dynamics marks the transition to the adaptation/resolution phase of macrophage infection, with consequent dampening of M1 polarization, as described in M. tuberculosis-infected BMDMs from C57BL/6 mice (16). Starting from the adaptation/resolution phase, M. tuberculosis shows improved survival and/or growth in M. tuberculosis-infected BMDMs from C57BL/6 mice (32). Thus, the macrophage response to M. tuberculosis infection appears to reveal a similar two-phase “defending” and “adaptation/resolution” process (Fig. 4), as seen in THP-1 cells exposed to LPS or proinflammatory cytokines (136) and in BALB/c BMDMs infected by Leishmania infantum (46).

Studies on the metabolic characteristics of the adaptation/resolution phase of macrophages during M. tuberculosis infection are lacking in the published literature. In THP-1 cells activated by TLR-4 signaling, increased glucose uptake and oxidation during the early inflammation phase (up to 8 h), as measured by d-[6-^14^C] glucose labeling, decreased, and glucose oxidation remained low at 24 h of the adaptation phase, which is consistent with the transition to an oxidative metabolism-dominated metabolic state in mitochondria (136). Similarly, during BALB/c BMDM infection by L. infantum, the bioenergetics profile of the early response is marked by enhanced glycolytic capacity (measured by increased extracellular acidification rate [ECAR]) and increased levels of key glycolytic enzymes) and by low mitochondrial metabolism (measured by the mitochondrial oxygen consumption rate [OCR]). At the late stage of the infection (24 h postinfection), the metabolic state of infected murine macrophages transitions to oxidative phosphorylation with increased expression of PGC-1α, which promotes mitochondrial biosynthesis, as evidenced by an increase in the mitochondrial DNA/nuclear DNA ratio and in expression of nuclear genes encoding mitochondrial complexes, such as *Ddufa* (complex I) and *Cox4* (complex IV) (46). A recent study on the characterization of bioenergetic metabolism by extracellular flux analysis in human primary macrophages and THP-1 cells indicated that, unlike nonpathogenic mycobacteria or dead mycobacteria, M. tuberculosis uniquely decelerates both glycolysis and oxidative phosphorylation, thereby entering a state of metabolic quiescence (137). Specifically, M. tuberculosis infection reduces the glycolytic proton efflux rate (glycoPER) of macrophages, which is derived by subtracting the mitochondrial proton efflux rate from the total proton efflux rate and which presents a more accurate measurement of glycolytic acidification/activity (137). The decelerated bioenergetic metabolism in macrophages during M. tuberculosis infection is also consistent with results of ^13^C-tracer experiments in the same study showing reduced total ^13^C incorporation into glycolytic intermediates and reduced secretion of lactate and pyruvate in the supernatant fluid of M. tuberculosis-infected human primary cells and THP-1 cells (137). These findings appear to contradict the observations from other experimental systems discussed above, such as the murine models, which showed increased glycolysis upon macrophage activation. This discrepancy is probably associated with the differing types of macrophage used and the different time points for cell harvesting and analysis post-M. tuberculosis infection. Importantly, both the flux analysis and the ^13^C-tracer experiments described in reference 137 were carried out 18 to 24 h after infection, which is beyond the early phase of metabolic changes occurred at 4 to 8 h postinfection, as discussed above. Therefore, the data reported in reference 137 likely represent the bioenergetic profile of the transition to or the adaptation/resolution phase of the macrophage response, which is also consistent with the increased mitochondrial dependency of macrophages on fatty acids, as observed in same study (137), and with observations of our transcriptome profiling analysis.

## SIRTUIN(S) AS MEDIATOR BETWEEN METABOLISM AND INFLAMMATION

A growing body of evidence indicates that sirtuins, which function through epigenetic modulation (138), are the main molecular actors mediating the relationship between innate immunity and cellular bioenergetics (139, 140). Sirtuins are a seven-member family of highly conserved, NAD^+^-dependent proteins having deacetylase activity (141). Early studies showed that the antagonistic cross talk between the NF-κB and SIRT1 signaling pathways plays an essential role in determining the inflammation state and energy supply at different stages of immune cell activation (142). NF-κB signaling drives the proinflammatory phenotype and glycolytic metabolism through cross talk with HIF-1α (143). In contrast, SIRT1 promotes anti-inflammatory responses and resolution of inflammation by inhibiting NF-κB signaling through deacetylating the NF-κB complex (144) and by supporting mitochondrial biogenesis and oxidative metabolism through reciprocal cross talk with the energy-sensing enzyme AMP-activated protein kinase (AMPK) (145). Several emerging lines of evidence also support the idea of the roles of SIRT6 in anti-inflammation and inhibition of glycolysis. SIRT6 attenuates NF-κB signaling by directly interacting with the NF-κB RelA subunit and by deacetylating histone H3K9 at the NF-κB target gene promoters (146). SIRT6 also serves as an important regulator of glucose metabolism and glycolytic flux by inhibiting the transcription of Warburg effect genes through directly binding and deacetylating histone H3K9 in their promoter regions and by serving as a corepressor for the transactivation of HIF-1 (147, 148). The idea that SIRT6 inhibits glucose metabolism and inflammation is further supported by the observations that *Sirt6* deficiency in mice causes early postweaning lethality from severe hypoglycemia (149). The same research group also found that a deficiency of *Sirt6* in immune cells leads to chronic liver inflammation in *Sirt6*^−/−^ mice; moreover, targeted deletion of *Sirt6* in macrophages and T cells results in enhanced glucose metabolism and their activation leading to the M1 phenotype and the Th1 response, respectively (150). In LPS-activated THP-1 cells, SIRT1 and SIRT6 coordinate a metabolic switch from glycolysis to fatty acid oxidation during the progression of the inflammation response (136).

*Sirt1* is marginally downregulated during both the early and adaptation phases of murine macrophage infection by M. tuberculosis. Decreased expression of *SIRT1/Sirt1* was also found in M. tuberculosis-infected THP-1 cells and in mouse lungs, and its activation contributed to control of infection by the host cells (151). SIRT1 expression appears to correlate negatively with the severity of TB disease, as peripheral blood cells of active TB patients have lower SIRT1 mRNA levels than those of healthy and latent TB individuals and those of the same TB patients before chemotherapy (151). Given the role of SIRT1 in promoting mitochondrial oxidative metabolism and restoring metabolic homeostasis of the M2 response (142, 152), it is conceivable that the benefit to the host of its activation is that of its limiting the potential damage caused by prolonged hyperinflammation during the chronic stage of lung infection. However, finding a similar protective effect of SIRT1 activation during the early phase of macrophage infection (151) is surprising, given that SIRT1 activation is expected to deacetylate RelA/p65 of NF-κB signaling and diminish the proinflammatory responses of infected macrophages (142, 152), which are required for infection control. Indeed, an opposite, detrimental role of *Sirt1* induction was found during L. infantum infection, in which the pathogen modulates an early aerobic glycolytic environment in BMDMs from BALB/c mice toward mitochondrial metabolism through the SIRT1/AMPK axis to increase its survival in host cells (46). Further studies are needed to identify the roles of SIRT1 in TB pathogenesis, especially given that certain activators, such as resveratrol and SRT1720 (151), are reported to interact with multiple unrelated targets and are not direct activators of SIRT1 (153). Intriguingly, in contrast to the results seen with *Sirt1*, our analysis revealed upregulation of *Sirt6* during both phases of murine macrophage infection. This observation suggests a novel aspect of M. tuberculosis pathogenesis, whereby M. tuberculosis modulates NF-κB signaling and the Warburg effect through SIRT6 signaling, thereby compromising the proinflammatory and antimicrobial responses of infected macrophages (Fig. 4). Pharmacological inhibition of SIRT6 has blood sugar-lowering effects that operate by enhancing glucose transporter expression and by stimulating glycolysis in wild-type and high-fat-diet-induced diabetic C57BL/6 mice (154). With validation of the anti-inflammatory role of SIRT6 and the availability of specific SIRT6 inhibitors (154–156), HDTs using these inhibitors can be tested in boosting bacterial clearance during the initial stages of M. tuberculosis infection. Once validated, this strategy may have paramount significance in treating TB patients during the early stages of reactivating disease, given that the majority of active TB cases derive from reactivation of a latent infection (10).

## CONCLUDING REMARKS

The similarity of the biphasic metabolic dynamics of macrophages in response to M. tuberculosis infection described above to the metabolic reprogramming occurring during macrophage activation in other settings indicates a common metabolic signature of the macrophage responses to diverse environmental signals. However, the novel insights into the mechanisms of TB pathogenesis are provided by the specific aspects of metabolic modulation induced by M. tuberculosis infection during M1 polarization. Future studies are warranted to dissect these molecular interactions, including signaling networks and metabolic pathways both upstream and downstream of these metabolic modulations. Given that macrophage functions are closely associated with the microenvironment of infection foci, it is essential to define the metabolic characteristics and immune properties of macrophages with regard to their spatial location within granulomas, the stage of infection, and infection outcome. It is equally important to define how macrophage metabolic reprogramming affects the metabolic state and function of T cells and the ability of host adaptive immunity to control M. tuberculosis infection. Such studies are expected to lead to the development of potential HDTs to enhance bacterial clearance and shorten the duration of antibiotic therapy.
